# Fostering the psychological wellbeing of children diagnosed with cancer: multidisciplinary insights in pediatric oncology

**DOI:** 10.3389/fpsyg.2025.1495969

**Published:** 2025-01-29

**Authors:** Giulia Perasso, Marco Romeo, Paola Coccia, Giulia Palego, Palina Perez-Duarte Mendiola

**Affiliations:** ^1^Department of Psychology, University of Genoa, Genoa, Italy; ^2^Associazione Nazionale Volontari Lotta contro i Tumori-ANVOLT, Milan, Italy; ^3^Pediatric Oncohematology Unit, Salesi Hospital, Ancona, Italy; ^4^Azienda Ospedaliero Universitaria delle Marche, Ancona, Italy; ^5^PEDAL Hub, University of Cambridge, Cambridge, United Kingdom

**Keywords:** psychology, psycho-oncology, pediatric oncology, cancer, play-based interventions, children

## 1 Introduction

In May-June 2024, the Italian associations of volunteers against cancer (Associazione Nazionale Volontari Lotta contro i Tumori, ANVOLT) promoted a five lesson-course entitled “Play and Therapy: Supporting Oncological Children through the Play-Based Approaches.” This interdisciplinary course was part of the project “Pathways of support for children and adolescents in an onco-hematological context: Assistance, Research, and Education” funded by the Ministry of Work and Social Politics. The initiative was moderated by a psychologist and freely available online for psychologists, psychotherapists, pediatricians, nurses, students (psychology and social work), teachers, hospital teachers, educators and hospital volunteers. Five lessons guided professionals in an in-depth reflection over the humanization of care for children affected by cancer. In the present paper, the main insights emerging from the course are synthetized, discussed and shared with the scientific community.

### 1.1 Focusing on the resources of children with cancer

Children diagnosed with cancer should increasingly be viewed as individuals possessing significant potential for wellbeing. The biopsychosocial model proposed by Engel (1978) emphasizes that health results from complex interactions between biological, psychological, and social factors. This approach, when applied to pediatric oncology, requires a shift from merely addressing the physical aspects of the disease to also considering the child's emotional and psychological wellbeing, as well as the impact on their family.

Evidence suggests that children with cancer often develop adaptive coping mechanisms that are dynamic rather than static. Research by Kupst et al. (1984) showed that oncological children's coping skills could become more constructive when supported by psychological support, regardless of the child's age at diagnosis or the stage of the illness. The family environment, including parents' coping styles, religion and culture, play a crucial role in shaping the child's psychological adaptation. Children whose parents maintain a cohesive and optimistic outlook are more likely to exhibit resilience and fewer psychological difficulties (Sanger et al., 1991). Social support from peers also contributes significantly to the wellbeing of children with cancer (Varni et al., 1994) even if, in specific conditions (e.g., weakened immune system), socialization opportunities may be limited. Additionally, a child's personal experiences, such as the loss of a grandparent or pet due to cancer, can influence how they perceive their own diagnosis and treatment (Pérez-Duarte Mendiola, 2020).

Plus, children with cancer often display greater optimism and emotional resilience than their healthy counterparts. Studies have shown these children tend to experience fewer depressive symptoms and demonstrate better overall psychological adjustment (Phipps, 2007; Phipps et al., 2014). Despite the numerous stressors associated with their illness, many oncology patients develop a heightened sense of life purpose, deeper familial connections, and an ability to prioritize life aspects meaningfully (Tomich and Helgeson, 2004; Gibbs et al., 2022). As healthcare professionals, recognizing these strengths can not only improve care strategies but also empower children and their families to navigate their journey with resilience and hope.

### 1.2 An evolutionary perspective

Understanding developmental psychology is crucial for healthcare professionals who aim to support children and adolescents in oncology and onco-hematology settings. Hospitalization exposes young patients to a range of mental health risks, including depression, withdrawal, regression, sleep disturbances, anxiety, and hypochondria, mainly because their familiar routines are disrupted (Chambers, 1993; Perasso et al., 2021). Age-specific and structured play activities, guided by specialists (Perasso and Ozturk, 2022) can provide these children with a sense of continuity with their pre-illness life (Romito et al., 2021) or offer an imaginative escape from reality (Tanaka et al., 2010). To fully grasp the importance of these interventions, healthcare professionals must understand key theories and principles of developmental psychology, which examines the systematic changes characterizing psychological evolution throughout an individual's life.

Developmental psychology doesn't focus solely on children but rather on the process of development itself, marking psychological milestones over time. It explores human abilities—cognitive, socio-emotional, executive, and moral—and how they evolve. For instance, Bronfenbrenner's (1979) ecological systems theory helps explain a child's development in a hospital setting by highlighting the various layers of influence. At the microsystem level, a child's experience is shaped by direct interactions with parents, caregivers, and medical staff (e.g., how a nurse explains a procedure or how a parent provides comfort during treatment). At the macrosystem level, broader societal and cultural factors, such as how cancer is portrayed in the media or societal attitudes toward illness, can also shape children's understanding of their diagnosis and influence their emotional response to hospitalization.

Piaget's (1952) theory of cognitive development is also relevant, highlighting how children assimilate information based on their developmental stage, such as the sensorimotor stage (birth to 2 years), preoperational stage (2–7 years), concrete operational stage (7–11 years), and formal operational stage (12 years and up). Piaget's model has been used to chronologically map health-related knowledge in children (Koopman et al., 2004). For instance, children in Piaget's preoperational stage might perceive illness as punishment for bad behavior, requiring healthcare professionals to provide reassurance and age-appropriate explanations (Kanizsa, 2013). Additionally, Vygotsky's (1978) concept of “scaffolding” is valuable in this context, emphasizing the need for healthcare professionals to offer the right level of support, neither overwhelming the child nor leaving them to struggle alone (Lambert et al., 2011).

### 1.3 Hospitalization as trauma

All the specialists working with oncological or onco-hematological children should recognize the traumatic potential of the experience of hospitalization and medical treatments. For children, hospitalization involves significant disruption to their familiar routines, environments, and activities, especially play, which is critical for their development (Favara-Scacco et al., 2001).

Children's fears related to hospitalization often stem from concerns about medical procedures, pain, death, mutilation, uncertain diagnoses, separation from parents, and a perceived loss of control and safety (Sahler et al., 2002; Svavarsdottir, 2005; Coyne, 2006). Despite the focus on medical treatments to counteract or mitigate physical symptoms, the psychological impact of hospitalization is frequently underestimated (Koukourikos et al., 2015). The disruption of a child's routine can result in symptoms such as anxiety, depression, phobias, and regressive behaviors (Chambers, 1993). Integrating play into the hospital experience is therefore crucial for maintaining psychological wellbeing.

Play can help normalize the hospital experience by providing continuity with pre-diagnosis life and offering a distraction from stress (Perasso et al., 2021). Properly trained professionals can employ play-based methods to explain medical procedures and facilitate emotional expression, which can reduce anxiety and promote a sense of normalcy and control (Pérez-Duarte Mendiola, 2024). By involving children in their care and using play as a therapeutic tool, these specialists help address the power imbalance inherent in healthcare settings (Bricher, 2000), leading to improved cooperation. It is important to notice that the COVID-19 pandemic has added layers of complexity to pediatric care, emphasizing the need for a play-based approach to mitigate additional stressors and support children's emotional wellbeing during hospitalization (Tsamakis et al., 2020; Perasso et al., 2023a).

### 1.4 Play is something serious

In pediatric healthcare, the role of play cannot be underestimated, and for evidence-based practice it is crucial to recognize that this is a specialized field requiring dedicated expertise. Play is a fundamental aspect of pediatric care that involves more than just recreational activities. It requires specific knowledge and targeted intervention: (1) normative play—providing a sense of familiarity and continuity to children's daily lives; (2) therapeutic play—to process complex emotions related to treatment and hospitalization; and (3) medical play—tools that replicate medical equipment and procedures, aiming to reduce fear (Sutton-Smith, 1999; Drewes, 2006; Cameron and Patte, 2018). Article 31 of the UN Convention on the Rights of the Child emphasizes that play is a fundamental right, highlighting its essential role in normal development and emotional wellbeing (UNICEF, 1989). Play serves as a critical tool for assessing a child's psychological and physical development, monitoring disease progression, and providing relief during hospitalization (Koukourikos et al., 2015). The International Classification of Functioning, Disability, and Health for Children and Youth (ICF-CY) reinforces the importance of play, especially for children with health conditions or disabilities, by integrating it into educational and rehabilitative processes (Cajola and Rizzo, 2014).

Evidence supports the efficacy of play-based interventions in reducing negative emotions such as anxiety and stress in hospitalized children, thereby enhancing their coping skills and pain management (Gill, 2010; Moore et al., 2015; Perasso et al., 2021). However, it is important to acknowledge that the implementation of play interventions is not without its challenges. One significant limitation is the variability in how play is integrated into healthcare settings (Goh et al., 2019; Gjærde et al., 2021). Additionally, the effectiveness of play-based interventions may be difficult to measure and influenced by different factors (e.g., child's individual needs, severity of their medical condition, and the availability of resources and trained personnel) (Perasso et al., 2021).

Health Play Specialists (HPS) and Certified Child Life Specialists (CCLS), working in the UK and the US, respectively, with their practice rooted in Anna Freud's research and psychodynamic theories (Plank, 1962; Bergmann, 1965), are trained specifically to develop and implement play programs tailored to pediatric patients (Hart-Spencer and Griffiths, 2015). Their training involves extensive study in psychological or pedagogical fields, typically requiring at least 3 years of formal education (Lookabaugh and Ballard, 2018; Metzger et al., 2013). Their role includes understanding medical procedures, facilitating emotional expression, and promoting coping strategies, which is essential for bridging the gap between medical treatment and emotional support (Harvey, 1984). Empirical evidence highlights the effectiveness of play-based approaches in managing various medical conditions, including chronic illnesses, severe burns, long-term hospitalizations, and end-of-life care by improving overall health outcomes and pain management (Li et al., 2016; Elliott, 2023).

### 1.5 Play-based interventions, in the light of technology and gender stereotypes

In addressing pediatric oncology patients, healthcare professionals must recognize the evolving role of technology and socio-anthropological considerations in play-based interventions. As mHealth applications increasingly integrate traditional healthcare with digital technologies, they offer innovative methods that use play to actively engage children diagnosed with cancer in their treatment (e.g., Pain Buddy, see Fortier et al., 2016; Cherry, see Berntsen and Babic, 2013; AMICO H, see Perasso et al., 2023b). The World Health Organization describes mHealth (i.e., Mobile Health) as a blend of conventional healthcare practices with communication technologies, which has become feasible due to the widespread availability of wireless connections (Istepanian and Lacal, 2003).

Research across different countries (Perasso, 2023) mHealth applications, developed through collaboration between healthcare researchers and computer engineers (Nguyen et al., 2016), provide critical tools for distraction, psychoeducation, and support in pediatric care (Coelho and Reis, 2021). These applications harness gamification principles—using game-like elements such as points and challenges—to enhance children engagement and motivation with their treatment (Zhou et al., 2019). Evidence supports the efficacy of mHealth in various aspects of pediatric care, including pain management and surgical procedures (Richardson et al., 2020). However, developing engaging and effective mHealth applications for pediatric patients remains a significant challenge (Zhou et al., 2019).

In addition to technological advancements, the socio-anthropological impact of gender of play must be considered. Margaret Mead's (1935) work highlights how social conditioning influences gender roles and behaviors from an early age. The gendering of play is evident in toy marketing, where products are often divided into gender-specific categories, reinforcing stereotypes (Carta et al., 2020). This dichotomy impacts how children perceive their roles and abilities, potentially limiting their self-expression and coping mechanisms. Healthcare professionals must be aware of these socio-cultural dimensions when implementing play-based interventions. For instance, gendered toys and narratives (Abbatecola and Stagi, 2020) can reinforce limiting stereotypes, such as portraying girls as passive or boys as emotionally stoic. Such portrayals may affect a child's coping strategies and emotional expression, especially in the context of serious illness.

## 2 Conclusion

The recent ANVOLT course, “Play and Therapy: Supporting Children with Cancer through Play-Based Approaches,” highlighted key aspects of meeting the needs of young cancer patients. Central to this training is the recognition that these children possess significant strengths and resilience, which can be harnessed through informed therapeutic practices. Understanding these strengths requires a solid foundation in developmental psychology, which helps professionals comprehend how illness and treatment affect children at various developmental stages. The course emphasized that play-based interventions are crucial for mitigating the trauma associated with hospitalization and medical treatments. Structured play provides children with a sense of continuity and normalcy, which is vital for their emotional wellbeing during such disruptive times. Additionally, the course highlighted the importance of adapting play to the contemporary world. mHealth applications offer innovative digital play tools that enhance patient empowerment, while understanding the need to overcome gender stereotypes in play may help creating more inclusive interventions. Incorporating these insights—rooted in developmental psychology, expert training, and awareness of the contemporary world —enables healthcare professionals to deliver comprehensive care to children diagnosed with cancer and their families.
